# A Prospective Study on the Relationship Between Driving and Non-occupational Computer Use With Risk of Dementia

**DOI:** 10.3389/fnagi.2022.854177

**Published:** 2022-05-16

**Authors:** Hikaru Takeuchi, Ryuta Kawashima

**Affiliations:** ^1^Division of Developmental Cognitive Neuroscience, Institute of Development, Aging and Cancer, Tohoku University, Sendai, Japan; ^2^Smart Aging Research Center, Tohoku University, Sendai, Japan; ^3^Department of Advanced Brain Science, Institute of Development, Aging and Cancer, Tohoku University, Sendai, Japan

**Keywords:** driving, computer use, dementia, sedentary activity, prospective

## Abstract

Sedentary behaviors have been associated with the risk of dementia in older adults. Whether driving and computer use are associated with the risk of dementia in older adults is an important research question. The participants of a longitudinal cohort study that included European middle- and old-aged adults at the baseline (2006–2010) who had not been diagnosed with dementia before 5 years after the baseline and had not died within 5 years after the baseline were followed up (until 2018) and analyzed. The associations between driving and non-occupational computer use time measured by the questionnaire at the baseline and incident dementia 5 years after the baseline were analyzed after correcting for confounding variables. Each analysis included approximately 370,000 participants and 1,000 cases. According to Cox proportional hazard models that divide subjects into four groups of habit duration levels [(a) 0 h; (b) less than 1 h, 1 h; (c), 2 h, 3 h; (d) 4 h or more, per/day)], the group with 0 h < driving time ≤1 h at the baseline exhibited a significantly lower risk of incident dementia than the other groups. In addition, in the analysis of non-occupational computer use duration, the 0 h group exhibited a significantly higher risk than the other groups. Our results indicate that different sedentary behaviors have different associations with dementia risk over time and have no simple dose–response relationship with dementia risk. The sedentary behavior risk assessments must consider these factors.

## Introduction

Automobiles are becoming an essential mode of transportation for older adults. Aging and driving are intertwined in many ways. Driving performance declines during the aging process (Wood, 1998) and in the early stages of dementia (Duchek et al., 2003). Following the onset of dementia, most people stop driving (Adler and Kuskowski, 2003). Sedentary behaviors, such as driving, increase the risk of dementia (Yan et al., 2020) and cardiovascular diseases (Pandey et al., 2016).

Furthermore, computer use is now a significant part of older adults’ cognitive activities. The sedentary behaviors that do not distinguish contents of the sedentary behaviors and longer TV viewing length have been associated with lower cognitive performance and longitudinal cognitive decline, as well as the risk of developing incident dementia (for a review, see Falck et al. (2017)). Consistently, the randomized controlled trials have shown the efficacy of physical activity on cognitive functions (de Almeida et al., 2020). Using computers for most of the day is frequently categorized as a “sedentary behavior.” However, previous cross-sectional and longitudinal studies have reported that the effects of “sedentary behaviors” on cognitive changes in aging adults are not unitary (Bakrania et al., 2018; Wanders et al., 2021). Wanders et al. (2021) revealed that while higher work-related sedentary times and computer use sedentary times were associated with better cognitive functions, the leisure sedentary time was not associated with cognitive functions. Similarly, Tun and Lachman (2010) revealed that greater computer use was associated with better executive function in a cross-sectional study. Bakrania et al. (2018), using middle- and old-aged adults, revealed that greater television watching and driving were linked to a deterioration in multiple cognitive functions, while greater non-occupational computer use time was associated with higher retention in many cognitive functions in a longitudinal study. In addition, Slegers et al. (2012) reported an association between the computer use habits (habits of computer use in general) in older adults and subsequent better cognitive changes in a longitudinal study. Finally, a prospective study investigated the associations of mobile phone use and Internet use with the risk of dementia over time and found that mobile phone use was associated with the risk of incident dementia (Almeida-Meza et al., 2021).

As described here, the previous studies have evaluated the effects of sedentary behaviors as a whole, comprising various cognitive activities, the use of mobile phones on subsequent incident dementia, or the effects of each type of sedentary behavior on longitudinal cognitive changes. However, no previous studies have investigated whether representative sedentary behaviors of driving habits and non-occupational computer use alone are associated with the subsequent onset of dementia, which is the present study’s aim.

For this study, we used a dataset from the UK Biobank of 500,000 middle-aged and older persons to examine whether the baseline driving and non-occupational computer use length are associated with the changes in the risk of developing dementia after 5 years following corrections for several potential confounding factors. We hypothesized that less driving and greater non-occupational computer use are associated with a lower risk of incident dementia over time. Our hypothesis is based on the findings that different sedentary behaviors have different correlates (Wanders et al., 2021), and that less driving and greater non-occupational computer use are related to favorable longitudinal changes in cognitive outcomes (Bakrania et al., 2018).

## Materials and Methods

### Participants

The UK Biobank provided the dataset used for this study, which was obtained from a prospective cohort study of a middle-aged population in the United Kingdom, whose procedures have been described elsewhere^1^. The North–West Multi-center Research Ethics Committee approved these experiments, and each participant provided written informed consent. The participants accessed one of the 22 assessment sites in the United Kingdom for data collection, and the baseline data were received from 502,505 participants. Our analysis included data from this first assessment (2006–2010). We conducted each analysis using data from all the participants for whom valid data for all independent and dependent variables were available. The descriptions in this subsection are largely reproduced from our previous study using the same methods (Takeuchi and Kawashima, in press).

### Assessment of Computer Use and Driving Habits

Computerized self-report questionnaires were used to collect data on lifestyle patterns related to non-occupational computer use and driving. The following questions were used to assess the time spent driving and using the computer: “In a typical day, how many hours do you spend driving?” and “In a typical day, how many hours do you spend using the computer? (Do not include using a computer at work.)” The answers were “less than an hour a day” or any integer value between 0 and 24. We distinguished an answer of “less than an hour a day” from the answer “0 (hours).” The question was asked twice to those who reported more than 6 h/day.

The responses were categorized into four groups as follows: (a) 0; (b) “less than an hour a day” or 1 h; (c) 2 h or 3 h; and (d) 4 h or more and the responses were used in the statistical analyses.

### Sociodemographic and Lifestyle Measurements as Covariates

We used self-reported gender data for this study. From the UK Biobank database, the neighborhood-level socioeconomic status at recruitment (cov1), education level at recruitment (cov2), household income (cov3), current employment status (cov4), metabolic equivalent of task hours (cov5), the number of people in the household (cov6), height (cov7), body mass index (BMI) (cov8), self-reported health status (cov9), duration of sleep (cov10), systolic blood pressure (cov11), current alcohol drinking level (cov12), current tobacco smoking level (cov13), race (cov14), diagnosis of diabetes, heart attack, angina, stroke, cancer, and other severe medical conditions (cov15–20), driver jobs (cov21), and visuospatial memory task performance (performance worse than 2 standard deviation (SD) were excluded) (cov22) were extracted or calculated and included as covariates together with age and sex. Refer to the Supplementary Material section for additional information.

Visuospatial memory performance was chosen as a measure for baseline cognitive function because it involves complex cognitive activities and memory processes, and the data were available for most participants. There are other types of complex cognitive measures in the UK Biobank data, but those test scores were unavailable for most participants as far as we are aware.

### Statistical Data Analyses

We used Predictive Analysis Software, version 22.0.0 (SPSS Inc., Chicago, IL, United States; 2010), to conduct statistical analyses. We employed Cox proportional hazards models to investigate the relationship of daily non-occupational computer use and driving habits with the risk of all-cause dementia over time, as previously described (Lourida et al., 2019). All-cause dementia was determined using hospital inpatient records and connections to death registry data. For more information, see Supplementary Material. This method of determining dementia was taken from a representative study that assessed the lifestyle with risk of incident dementia over time using the UK Biobank data (Lourida et al., 2019) as well as our previous study (Takeuchi and Kawashima, in press). The descriptions in this subsection are largely reproduced from our previous study using the same methods (Takeuchi and Kawashima, in press).

Participants with (a) self-reported dementia or Alzheimer’s disease or cognitive impairment without a diagnosis of all-cause dementia in either hospital inpatient records or death register data, (b) subjects already diagnosed with dementia at the baseline or within 5 years after the baseline, (c) those who died within 5 years after the baseline, and (d) those with visuospatial memory performance lower than 2 SD were excluded from the analyses. The observation period started with each participant’s first assessment visit and continued until the event or on 28 February 2018. For each analysis, sex and age at the baseline, and cov1–22 values were all used as covariates. The subjects who were diagnosed within 5 years after the baseline were excluded because we would like to remove the possibility that pre-clinical conditions of dementia affect relevant behaviors as much as possible, which has often been done in prospective studies of dementia related to lifestyles (Luojus et al., 2017).

The results of analyses with a threshold of *p* < 0.025 (two-tailed) in statistical values of the existence of overall group difference were considered significant, and *post hoc* comparisons among each group were conducted.

## Results

### Basic Baseline Data

Table 1 shows the baseline psychological variables of the participants included in the analysis. In the analyses of driving length and non-occupational computer use length, the data from 367,376 participants (173,573 males) and 369,036 participants (174,217 males) without incident dementia and 1,015 participants (612 males) and 1,014 participants (611 males) who had incident dementia later than 5 years after baseline were included, respectively. The mean age of the former group was 55.9 (SD: 8.1) and that of the latter group was 64.1 (SD: 5.0) in both analyses.

**TABLE 1:** Baseline characteristics of participants with and without incident dementia later.

	Analysis of driving length		Analysis of non-occupational computer use length	
		
	No incident dementia (*n* = 367,376)	Incident dementia (*n* = 1,015) Mean	No incident dementia (*n* = 369,036) (SD)	Incident dementia (*n* = 1,014)
Age	55.9 (8.1)	64.1 (5)	55.9 (8.1)	64.1 (5)
Townsend deprivation index	−1.44 (2.99)	−1.02 (3.2)	−1.44 (2.99)	−1.03 (3.2)
Education length	14.4 (5.1)	12.7 (5.2)	14.4 (5.1)	12.7 (5.2)
MET*	31.9 (35.6)	31.8 (39.6)	31.9 (35.6)	31.6 (39.2)
BMI	27.3 (4.7)	27.5 (5)	27.3 (4.7)	27.5 (5)
Height	169.1 (9.2)	168.6 (9.3)	169 (9.2)	168.6 (9.3)
Systolic BP	137.3 (18.5)	143.7 (20.5)	137.3 (18.5)	143.7 (20.6)
Visuospatial memory (errors)	3.64 (2.43)	4.34 (2.78)	3.64 (2.43)	4.34 (2.77)
		Number	(percent)	
Male number	173,573 (47.25%)	612 (60.3%)	174,217 (47.21%)	611 (60.26%)
**Household income** (a) Less than £18,000 (b) £18,000 to £30,999 (c) £31,000 to £51,999 (d) £52,000 to £100,000 (e) Greater than £100,000	75,922 (20.7%) 92,112 (25.1%) 98,672 (26.9%) 79,294 (21.6%) 21,376 (5.8%)	462 (45.5%) 301 (29.7%) 154 (15.2%) 80 (7.9%) 18 (1.8%)	76,570 (20.7%) 92,558 (25.1%) 99,000 (26.8%) 79,500 (21.5%) 21,408 (5.8%)	458 (45.2%) 304 (30%) 154 (15.2%) 80 (7.9%) 18 (1.8%)
Currently employed	228,970 (62.33%)	247 (24.33%)	229,684 (62.24%)	247 (24.36%)
**Household number** (a) 1 (b) 2 (c) 3 (d) 4≤	68,167 (18.6%) 166,683 (45.4%) 58,438 (15.9%) 74,088 (20.2%)	265 (26.1%) 601 (59.2%) 99 (9.8%) 50 (4.9%)	68,526 (18.6%) 167,505 (45.4%) 58,683 (15.9%) 74,322 (20.1%)	261 (25.7%) 605 (59.7%) 99 (9.8%) 49 (4.8%)
**Overall health (4 levels)** (a) Poor (b) Fair (c) Good (d) Excellent	13,232 (3.6%) 71,207 (19.4%) 216,933 (59%) 66,004 (18%)	122 (12%) 282 (27.8%) 512 (50.4%) 99 (9.8%)	13,323 (3.6%) 71,587 (19.4%) 217,928 (59.1%) 66,198 (17.9%)	123 (12.1%) 282 (27.8%) 510 (50.3%) 99 (9.8%)
**Sleep duration** (a)≤4 h, (b) 5 h or 6 h, (c) 7 h or 8 h, (d) 9 h≤	3,223 (0.9%), 84,064 (22.9%), 254,138 (69.1%), 25,951 (7.1%)	20 (2%), 225 (22.2%), 636 (62.7%), 134 (13.2%)	3,248 (0.9%), 84,513 (22.9%), 255,162 (69.1%), 26,113 (7.1%)	21 (2.1%), 228 (22.5%), 631 (62.2%), 134 (13.2%)
**Alcohol consumption (unit)** (a) 0, (b) 0 < *x* ≤ 14, (c) 14 < *x* ≤ 28 (d) 28 < *x*	24,948 (6.8%), 176,610 (48%), 88,109 (24%), 77,709 (21.1%)	126 (12.4%), 458 (45.1%), 218 (21.5%), 213 (21%)	25,092 (6.8%), 177,578 (48.1%), 88,406 (24%), 77,960 (21.1%)	125 (12.3%), 458 (45.2%), 217 (21.4%), 214 (21.1%)
**Current smoking level (3 levels)** (a) No (b) Only occasionally (c) On most or all days	330,281 (89.9%) 10,177 (2.8%) 26,918 (7.3%)	888 (87.5%) 36 (3.5%) 91 (9%)	331,805 (89.9%) 10,216 (2.8%) 27,015 (7.3%)	886 (87.4%) 36 (3.6%) 92 (9.1%)
Ethnicity (non-white)	15,567 (4.24%)	23 (2.27%)	15,714 (4.26%)	24 (2.37%)
Diabetes	17,048 (4.64%)	138 (13.6%)	17,143 (4.65%)	138 (13.61%)
Heart attack	7,471 (2.03%)	86 (8.47%)	7,514 (2.04%)	88 (8.68%)
Angina	10,049 (2.74%)	106 (10.44%)	10,121 (2.74%)	108 (10.65%)
Stroke	4,663 (1.27%)	62 (6.11%)	4,682 (1.27%)	61 (6.02%)
Cancer	28,469 (7.75%)	104 (10.25%)	28,609 (7.75%)	104 (10.26%)
Other serious medical conditions	71,762 (19.53%)	352 (34.68%)	72,159 (19.55%)	353 (34.81%)
Having driver jobs	813 (0.22%)	0 (0%)	819 (0.22%)	0 (0%)
Driving length (a) 0 h, (b) “less than an hour” or 1 h, (c) 2 h or 3 h, (d) 4 h≤	70,314 (19.1%), 226,184 (61.5%), 58,470 (15.9%), 12,40 8(3.4%)	30 30.4%), 52 51.8%), 153 (15.1%), 27 (2.7%)		
**Non-occupational computer use length** (a) 0 h, (b) “less than an hour” or 1 h, (c) 2 h or 3 h, (d) 4 h≤			89,207 (24.2%), 193,199 (52.4%), 64,250 (17.4%), 22,380 (6.1%)	456 (45%), 340 (33.5%), 174 (17.2%), 44 (4.3%)

*MET, metabolic equivalent of task hours (MET).

*Physical activity level.*

### Prospective Dementia Analysis

**FIGURE 1:**
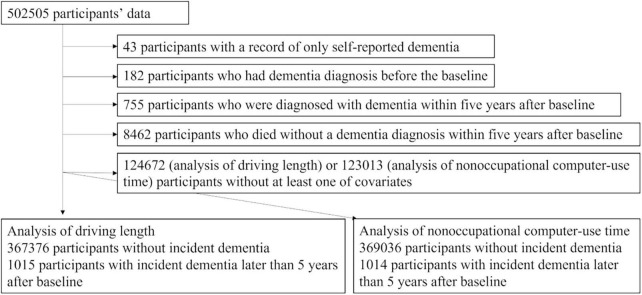
A flowchart of the study exclusion procedures.

There were 502,505 participants’ data in this study. From them, we excluded 43 participants with a record of only self-reported dementia or cognitive impairment, followed by 182 participants who had dementia diagnosis before the baseline and 755 participants who were diagnosed with dementia within 5 years after baseline. Then, we excluded 8,462 participants who died without a dementia diagnosis within 5 years after the baseline. Among these, we conducted studies using the samples of those who had all covariates, including visuospatial memory performance better than the worse 2σ. Figure 1 presents the flowchart.

**FIGURE 2:**
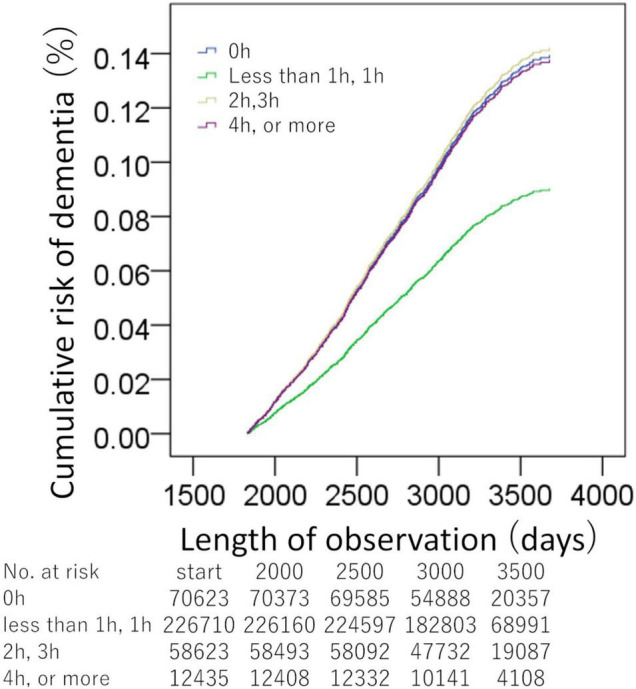
Standardized risks of incident dementia over time according to the daily driving time. Cox proportional hazards models were adjusted for potential confounding variables. There were significant differences between the group of less than 1 h, or 1 h, and the other groups.

The Cox proportional hazard models, which divided the subjects into four groups of habit duration levels [(a) 0 h; (b) less than 1 h, 1 h; (c) 2 h, 3 h; (d) 4 h, or more than 4 h] and which corrected potential confounding variables, were conducted for driving habit and non-occupational computer use, respectively. In the driving length analysis, over 368,391 person-years of follow-up [median (interquartile range) length of follow-up, 9.1 (8.4–9.7) years], there were 1,015 cases of incident all-cause dementia later than 5 years after the baseline. In the driving habit analysis, the group with less than 1 h, 1 h at the baseline (526 among 226,710 participants) showed a significantly lower risk of incident dementia than the group of 0 h (309 among 70,623 participants, HR: 1.544, 95% CI: 1.324–1.801, *p* = 3.07 × 10^–8^), the group of 2 h, 3 h (153 among 58,623 participants, HR: 1.574, 95% CI: 1.307–1.895, *p* = 0.000002), and the group of 4 h, or more (27 among 12,435 participants, HR: 1.525, 95% CI: 1.023–2.274, *p* = 0.038) (Figure 2). The *p*-value of the overall group difference was 2.19 × 10^–9^. The *p*-value for the trend of the four categories based on length was 0.518.

**FIGURE 3:**
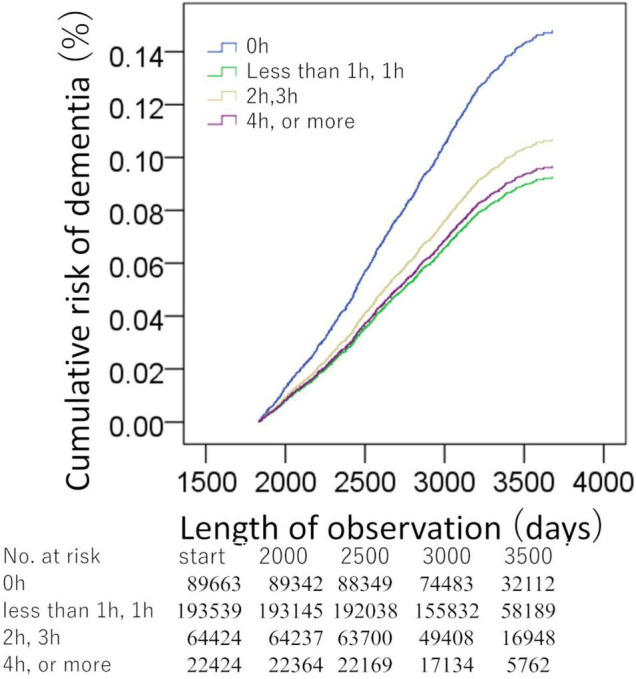
Standardized risks of incident dementia over time according to the daily non-occupational computer use duration. Cox proportional hazards models were adjusted for potential confounding variables. There were significant differences between the group of no non-occupational computer use time (*x* = 0 h) and the other groups.

In the analyses of non-occupational computer use length, over 370,050 person-years of follow-up [median (interquartile range) length of follow-up, 9.1 (8.4–9.7) years], there were 1,014 cases of incident all-cause dementia later than 5 years after baseline. In the analysis of the non-occupational computer use, the group of 0 h (456 among 89,663 participants) showed significantly higher risks than the group of less than 1 h, 1 h (340 among 193,539 participants, HR: 0.626, 95% CI: 0.538–0.728, *p* = 1.20 × 10^–9^), the group with 2 h, 3 h (174 among 64,424 participants, HR: 0.722, 95% CI: 0.601–0.869, *p* = 0.001), and the group of 4 h or more (44 among 22,424 participants, HR: 0.653, 95% CI: 0.476–0.897, *p* = 0.009) (Figure 3). The *p*-value of the overall group difference was 1.50 × 10^–8^. The *p*-value for the trend of the four categories based on length was 2.3 × 10^–5^.

**FIGURE 4:**
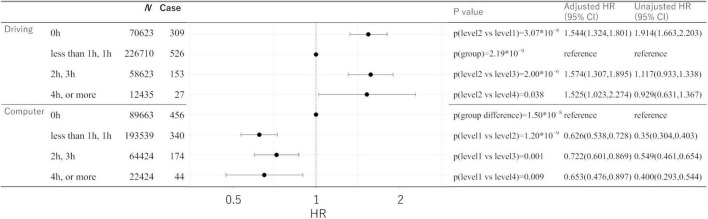
Statistical values and hazard ratios (95% CIs) for the associations between driving length, non-occupational computer use length, and incident dementia for more than 5 years after baseline in the UK Biobank data. Participants are categorized according to the length level at the baseline. “*p* (group)” indicates the *p*-values of the existence of the group difference among all the groups. 95% CI, 95% confidence interval; HR, hazard ratio.

Supplementary Table 1 presents the statistical values of the effects of the covariates in the two analyses. And Figure 4 presents statistical values of effects of driving length and non-occupational computer use length.

### Supplemental Analyses Based on Age Group

In the driving time analysis, 168 and 847 subjects who were younger and older than 60 at the baseline developed dementia 5 or more years after the baseline, respectively. For the non-occupational computer use time analysis, the numbers were 166 and 848, respectively.

Because the subjects younger than 60 years of age at the baseline would not reach the age at which dementia frequently occurs even after the follow-up period of this study, analyses excluding subjects younger than 60 years of age at the baseline were also performed.

The analyses excluding subjects younger than 60 years of age at the baseline revealed significant results for the same contrast sets as in the main analyses, except that in the analysis of non-occupational computer use, compared with the group of 0 h, the group of 4 h or more showed a statistical tendency toward higher risks (*p* = 0.080). The analyses excluding subjects older than 59 years of age at the baseline revealed significant results for the same contrast sets as in the main analyses, except that in the analysis of driving length, compared with the group of less than 1 h, or 1 h, the group of 4 h or more did not show significantly higher risks (HR: 1.232, 95% CI: 0.524–2.896, *p* = 0.633); in the analysis of non-occupational computer use, compared with the group of 0 h, group of 2 h or 3 h did not show significantly lower risks (HR: 0.919, 95% CI: 0.588–1.436, *p* = 0.710). Supplementary Table 2 presents the statistical values of these analyses.

### A Supplemental Analysis Including Both Driving Length and Non-occupational Computer Use Length as Covariates at Once

In the main analyses, we conducted two separate Cox proportional hazard models for the analyses of driving length and non-occupational computer use length. The driving length and non-occupational computer use length variables used in this study showed a significant positive correlation in this cohort (*r* = 0.092, *p* < 0.001; they were treated as continuous variables in this calculation). The driving length and non-occupational computer use length did not show correlations levels exceeding *r* > 0.25 when all variables were treated as continuous variables. Next, we evaluated the impact of including both driving length and non-occupational computer use length as covariates in one Cox proportional hazard model at once. This analysis revealed the significant results for the same contrast sets as in the main analyses, except that in the result of the driving, compared with the group of (b) less than 1 h, or 1 h, the group of (d) 4 h or more showed a statistical tendency toward higher risks (*p* = 0.120). Supplementary Table 2 presents the statistical values of these analyses.

### A Sensitivity Analysis Excluding Subjects With a Medical History or Cancer, Cardiovascular Disease, and Cognitive/Psychiatric Illness

In this study, we included several medical histories as covariates. Next, we conducted a sensitivity analysis excluding subjects with a medical history or cancer, cardiovascular disease, and cognitive/psychiatric illness.

In this analysis, we excluded subjects with a diagnosis of diabetes, heart attack, angina, stroke, cancer, and other severe medical conditions as specified by cov15–20 instead of including these as covariates (these were removed from covariate sets as well). We also excluded subjects with a self-reported medical diagnosis of schizophrenia and depression based on the UK Biobank item ID: 20544.

This analysis revealed significant results for the same contrast sets as in the main analyses, except that in the result of driving, compared with the group of (b) less than 1 h, or 1 h, the group of (d) 4 h or more showed a statistical tendency of higher risks (*p* = 0.102). Supplementary Table 2 presents the statistical values of these analyses.

### Supplemental Analyses Separating the Group of “Less Than an Hour” and 1 h

In this study, “less than an hour” was placed in the same category as 1 h when creating the four categories. This category implies that the respondents drive and use computers, but little. This is because it is unlikely that “less than an hour” would mean 0 h of no use when there are options of 0 h, 1 h, and less than 1 h of use. However, just to be sure, we conducted five-category analyses, distinguishing between less than 1 h and 1 h. These supplemental analyses were otherwise identical to the main analyses. The results showed that “less than an hour” and 1 h had similar properties.

The analysis of the driving length showed generally the same pattern of significant results as the main analysis, but the difference in risk between the group of 1 h and the group of 4 h or more failed to reach significance and only showed a tendency (*p* = 0.113). Other comparisons between the group of “less than an hour” or the group of 1 h and the other three groups showed significant differences as those of the main analysis.

In the analysis of non-occupational computer use length, the group of 0 h showed a significantly higher risk than the other four groups, as was the case in the main analysis.

## Discussion

We used a large data sample of middle- and old-aged adults in this study to evaluate the relationship between driving and non-occupational computer use habits at the baseline and the subsequent onset of dementia later than 5 years after the baseline, after correcting for several potential confounding variables. Our results were inconsistent with our hypothesis, and there was no simple dose–response relationship between driving habits, non-occupational computer use, and dementia risk over time. Instead, for driving habits, the group with little driving time (less than 1 h or 1 h/day) was characterized by a lower risk of dementia than the group with longer driving time (2 h, 3 h/day, 4 h, or more than 4 h/day) and the group with no driving habit (0 h/day). Regarding non-occupational computer use habit, the group with no computer use (0 h/day) had a higher risk of incident dementia over time than all the other groups, and we observed no dose–response relationship between non-occupational computer use time and dementia risk over time.

For driving, the group with little driving time (less than 1 h or 1 h/day) showed the lowest risk of incident dementia over time compared with the groups with no driving habit or longer driving time. As stated in the Introduction, driving ability deteriorates as dementia develops (Duchek et al., 2003). Sedentary behaviors and driving cessation are also characteristics of dementia (Adler and Kuskowski, 2003; van Alphen et al., 2016). A meta-analysis revealed a strong relationship between sedentary behaviors and the subsequent onset of dementia (Yan et al., 2020), but different sedentary behaviors are known to have different cognitive correlates (Bakrania et al., 2018; Wanders et al., 2021). This study advances these earlier studies by showing that, among those who keep driving, shorter driving distances are related to a lower risk of dementia over time, even after controlling for BMI and physical activity levels. Furthermore, we also advanced previous studies by showing that no driving habit is also a risk factor for dementia risk over time, and that driving time has no simple linear dose–response relationship with dementia risk over time. Whether the present association reflects causality [e.g., cognitive components required in driving prevent cognitive decline, and/or longer driving leads to thrombosis (Lippi and Favaloro, 2018), which is also associated with stroke (Bembenek et al., 2011)] or reverse causation (pre-clinical conditions of dementia lead to wandering using cars, getting lost during driving, lower driving ability, and cessation of driving) should be confirmed in future studies.

Regarding non-occupational computer use, no use was associated with an increased risk of dementia over time, but no other dose–response relationship existed. This is partly consistent with a previous study that showed a lack of non-occupational computer use and subsequent increased cognitive decline in aging adults (Slegers et al., 2012). Additionally, a longitudinal study showed an association of non-occupational computer use with a beneficial change in executive functioning (Hartanto et al., 2020), in contrast to the cross-sectional findings that children with a high amount of non-occupational computer use were associated with weaker performance in test measuring shifting and flexibility of attention (Syväoja et al., 2014). Future research should determine whether the pre-clinical stage of dementia induces lack of use of non-occupational computers or whether lack of use of non-occupational computers induces increased risk of dementia over time. Regardless, it is inappropriate to classify prolonged non-occupational computer use as a risk factor for dementia over time as a part of “sedentary behaviors.”

This study has several limitations. Even with prospective longitudinal designs, it is difficult to exclude reverse causation. Dementia’s neuropathological processes begin 20 years before symptoms appear (Bateman et al., 2012). Although we corrected for cognitive functions at baseline, the lack of driving or non-occupational computer use may indicate uncorrected incapability rather than habit. Therefore, an increase in the risk associated with not driving or using a computer may need to be interpreted cautiously. Future intervention studies will need to confirm causality. Additionally, in the UK Biobank, computer use duration is defined as the non-occupational computer use time. Therefore, the total duration of computer use could not be determined in this study, potentially blurring the dose–response relationship between computer use and the risk of incident dementia over time. Another limitation of this study is that the measures of driving and computer use length in the UK Biobank are not independently validated, and they are self-reported measures. Further, although the analyses were adjusted for various covariates, unmeasured confounding factors must remain more or less. In addition, in this study of the UK Biobank, we relied on the medical record of dementia in the large cohort, as was the case with previous representative studies of dementia using UK Biobank data (Lourida et al., 2019). The diagnosis of dementia is not actively conducted, and potential dementia cases might be missed more or less. The baseline age of the subjects included in this study’s analysis, alongside the observation period of the analysis, gives an age of 65 years. In Europe, the cumulative incidence of dementia in the 65–69 age group is reported to be about 1% (Lobo et al., 2000). Combined with the excluded subjects (self-reported), the current study found that 0.4% of the subjects had dementia at the end of the follow-up, which is not substantially different from previous estimates. Another limitation of the present study is the lack of control over the global cognitive measures. In this study, we controlled the baseline cognitive function using visuospatial memory performance. Most UK Biobank’s samples do not have data for more global cognitive measures at baseline, to our knowledge. However, data for non-verbal reasoning exist, but only minor parts of participants have data for this measure, and adding this variable results in cutting the number of participants with incident dementia into 215 and 217 in the present two analyses, which substantially prevents statistical power.

Previous studies have reported that sedentary behaviors are associated with an increased risk of incident dementia. This study evaluated the associations between representative sedentary behaviors—driving and non-occupational computer use habits—and the subsequent onset of dementia. Groups with little driving length showed the lowest risk compared with the group with no driving habit and a higher driving habit. Unlike driving habits, non-occupational computer use did not show such patterns, and regardless of the duration of computer usage, the group with non-occupational computer use showed a lower risk of dementia than the group with no non-occupational computer use. Our results indicate that sedentary behaviors have neither unilateral consequences nor a simple dose–response relationship with the subsequent onset of dementia. Sedentary behavior risk assessments must consider these factors.

## Data Availability Statement

Publicly available datasets were analyzed in this study. These data can be found here: UK Biobank, application 56726. Data are available upon the request to and approval from UK Biobank.

## Ethics Statement

Approval for these experiments was obtained from the North-West Multi-center Research Ethics Committee, and written informed consent was obtained from each participant. The project involving the use of human data of UK Biobank was reviewed and approved by the Ethics Committee of Tohoku University Medical Faculty.

## Author Contributions

HT conceptualized the study, preprocessed and analyzed the data, and wrote the manuscript. RK played a crucial role in obtaining relevant funding and supervising this study. Both authors contributed to the article and approved the submitted version.

## Conflict of Interest

The authors declare that the research was conducted in the absence of any commercial or financial relationships that could be construed as a potential conflict of interest.

## Publisher’s Note

All claims expressed in this article are solely those of the authors and do not necessarily represent those of their affiliated organizations, or those of the publisher, the editors and the reviewers. Any product that may be evaluated in this article, or claim that may be made by its manufacturer, is not guaranteed or endorsed by the publisher.

## Footnotes

1
https://www.ukbiobank.ac.uk/media/gnkeyh2q/study-rationale.pdf
