# Supplementary material for: A Prospective Study on the Relationship Between Driving and Non-occupational Computer Use With Risk of Dementia
Source: Front Aging Neurosci. 2022 May 16;14:854177. doi: 10.3389/fnagi.2022.854177 (PMC9149095; doi:10.3389/fnagi.2022.854177)
Supplement: Supplementary file 1 [file Data_Sheet_1.docx]

**Supplementary online material**

**Supplemental methods**

**Additional information on study subjects**

Adults between 40 and 69 years old who were registered with the National Health Service (NHS) and lived within 25 miles of the study’s evaluation sites were invited by email to participate in the UK Biobank. No exclusion criteria were applied for recruitment. The descriptions in this subsection were largely reproduced from another study that used the UK Biobank data ([Takeuchi and Kawashima, 2021](#_ENREF_7)).

**Details of sociodemographic and lifestyle measures**

The following descriptions in this subsection were largely reproduced from another study that used the UK Biobank data ([Takeuchi and Kawashima, 2021](#_ENREF_7)).

Cov1: Neighborhood-level socioeconomic status was measured using the Townsend index of material deprivation ([Townsend, 1987](#_ENREF_8)). Status was calculated based on the postcode of the subject’s address and represents a composite index of four postcode-level socioeconomic status variables: household overcrowding, unemployment, non-home ownership, and non-car ownership. A higher score implies a lower socioeconomic status. For this variable, only the value at recruitment was available and used for all analyses.

Cov2: The education level was based on self-reported data. Education level categories of participant choices were transformed into numerical values based on a previous study ([Okbay et al., 2016](#_ENREF_4)) as follows: “College or University degree” = 20 years; “A levels/AS levels or equivalent” = 13 years; “O levels/GCSEs or equivalent” = 10 years; “CSEs or equivalent” = 10 years; “NVQ or HND or HNC or equivalent“ = 19 years; “Other professional qualifications, e.g., nursing, teaching” = 15 years; “None of the above” = 7 years; and “Prefer not to answer” = missing. For this variable, only the value at recruitment was available, so it was used for all analyses.

Cov3: The household income was the self-reported total income (before taxes) received by the subject’s household. The available choices were <£18,000, £18,000 to £30,999, £100,000, £31,000 to £51,999, £52,000 to £100,000, >£100,000, “do not know,” and “prefer not to answer.” We converted these choices into ordinal variables between 1 and 5 (>£100,000 = 5) ([Shen et al., 2018](#_ENREF_6)) after excluding the answers of “do not know” and “prefer not to answer,” as elsewhere.

Cov4: The variable “current employment status” was used to describe the participants’ occupations. The responses to the variable were “In paid employment or self-employed,” “Retired,” “Looking after home and/or family,” “Unable to work because of sickness or disability,” “Unemployed,” “Doing unpaid or voluntary work,” “Full or part-time student,” and “None of the above.” Multiple responses were allowed. Subject responses were classified as either “In paid employment or self-employed” or not.

Cov5: The physical activity level was calculated from the recorded items from the International Physical Activity Questionnaire short form and converted into a single measure of total physical activity in the metabolic equivalent of task hours (MET). For more details, see ([Cullen et al., 2018](#_ENREF_1)).

Cov6: Participants were asked about the number of people in their household (including institutions such as care homes). Answers were assigned one of four variables: 1 (single person), 2 (two people), 3 (three people), and 4 (≥4 people), as elsewhere ([Sarkar et al., 2008](#_ENREF_5)).

Cov7,8: Body weight was measured using Tanita BC418MA scales. Height was measured using a Seca height measure. BMI was calculated from the measured height and weight.

Cov9: Participants were asked about their health status, with possible answers being excellent, good, fair, poor, and converted to values of 4, 3, 2, and 1, respectively, before input in the statistical analyses.

Cov10: Sleep duration was assessed with the item “About how many hours sleep do you get in every 24 h? (Please include naps.)” Responses were coded as integers and divided into four groups, (a) ≤4 h, (b) 5 h or 6 h, (c) 7 h or 8 h, (d) ≤9 h.

Cov11: Systolic blood pressure was measured using a digital BP monitor (Omron), or a manual sphygmomanometer when the digital monitor was unavailable. One or two readings were taken, and we used the average, as described in a previous study ([Veldsman et al., 2020](#_ENREF_9)).

Cov12: The current alcohol intake level was calculated as previously described ([Howe et al., 2019](#_ENREF_2)). Study participants were asked to describe their current drinking status (never, previous, current, prefer not to say), and estimate their current alcohol intake frequency (daily or almost daily, three or four times a week, once or twice a week, one to three times a month, special occasions only, never, prefer not to say). Individuals reporting a current intake frequency of ≥1–2 a week were asked to estimate their average weekly intake of a range of different alcoholic beverages (red wine, white wine, champagne, beer, cider, spirits, fortified wine). From these variables, we calculated the average intake of alcoholic units per week. This was derived by combining the self-reported estimated intake of different alcoholic beverages across all types. This calculation used the following measurement units for each of the five alcoholic drink types: measures for spirits, glasses for wines, and pints for beer/cider, were estimated to be equivalent to 1, 2, and 2.5 units, respectively. Individuals reporting a current intake frequency of “one to three times a month,” “special occasions only,” or “never” were assumed to have a weekly alcohol consumption volume of 0. Based on the obtained variable, four categories (a) 0, (b) 0 < x ≦ 14, (c) 14 < x ≦ 28 (d) 28 < x were generated.

Cov13: Participants were asked about their current tobacco smoking status. Possible answers were 1 (No), 2 (Only occasionally), and 3 (Yes, on most or all days) and were treated as a categorical variable. Responses of “prefer not to answer” were excluded.

Cov14: Ethnicity was self-reported, and possible effective answers were divided into white or other and analyzed.

Cov15–20: Participants were asked about the existence of a medical diagnosis of diabetes, heart attack, angina, stroke, cancer, and other serious medical conditions (item ID: 1049, 6150, 2453 2473). A dichotomized variable of existence for each condition was generated based on this answer.

Cov21: Visuospatial memory was measured using the “pairs-matching” task. In this test, participants were asked to memorize the positions of six card pairs and then match them from memory while making as few errors as possible. Scores on the pairs-matching test corresponded to the number of errors that participants made; therefore, higher scores reflected poorer cognitive functions.

Cov22: A dichotomized variable of the participant in jobs related to driving was created from the UK Biobank job coding data (Field 22601). The participant in either of the following lists at the time of the first visitation was coded as one, and the remainder were coded as 0: “refuse truck driver,” “refuse or rubbish truck driver,” “heavy goods vehicle (hgv) driver, lorry or truck driver, tanker driver, haulage driver,” “bus or coach driver, minibus driver,” “van driver, delivery driver, courier driver,” “taxi or cab driver, chauffeur, hearse driver,” “spreading machine driver, asphalt or tar-sprayer driver,” “agricultural machine driver, tractor driver,” “jcb driver, plant operator, bulldozer driver, caterpillar driver,” “fork-lift truck driver or operator,” “crane driver/operator, gantry operator, banksman (coalmine, salt works),” “agricultural machine driver, tractor driver,” “roadsweeper driver,” “mobile machinery driver,” “agricultural machine driver, tractor driver,” “jcb driver, plant operator,” “bus or coach driver, minibus driver,” “jcb driver, plant operator, pile driver,” “ambulance staff (not paramedic), ambulance driver, ambulance technician,” “train driver,” and “tram driver.”

**Determination of dementia**

To determine dementia of all causes, we followed the methods established in a previous study ([Lourida et al., 2019](#_ENREF_3)), with descriptions in this subsection mostly being reproduced from another study that used the UK Biobank data ([Takeuchi and Kawashima, 2021](#_ENREF_7)). All-cause dementia was determined based on hospital inpatient records containing data on admissions and diagnoses from the Hospital Episode Statistics for England, Scottish Morbidity Record Data for Scotland, and the Patient Episode Database for Wales. Additional cases were identified through death register data provided by the NHS Digital for England and Wales and the Information and Statistics Division for Scotland. Diagnoses were recorded using the International Classification of Diseases (ICD) coding system. Participants with dementia were identified as having a primary/secondary diagnosis (hospital records) or underlying/contributory cause of death (death register) using ICD-9 and ICD-10 codes for Alzheimer’s disease and other dementia classifications.

**Supplemental Table 1.** Statistical values of effects of covariates in main analyses

|  | analysis of driving length | | analysis of nonoccupatonal computer use length | |
| --- | --- | --- | --- | --- |
|  | P value  (uncorrected) | Adjusted HR  (95% CI) | P value  (uncorrected) | Adjusted HR  (95% CI) |
| age (1 increase) | 5.40*10^−98^ | 1.18(1.162–1.198) | 3.13*10^−94^ | 1.177(1.159–1.196) |
| Townsend deprivation index (1 increase) | 0.114 | 1.017(0.996–1.039) | 0.022 | 1.025(1.003–1.046) |
| Education length (1 year increase) | 0.247 | 0.992(0.98–1.005) | 0.840 | 0.999(0.986–1.012) |
| Household income (1 level increase) | 0.004 | 0.892(0.825–0.964) | 0.016 | 0.909(0.841–0.982) |
| MET (1 increase) | 0.819 | 1(0.998–1.002) | 0.595 | 1(0.998–1.001) |
| Household number (1 level increase) | 0.984 | 0.999(0.911–1.095) | 0.989 | 0.999(0.911–1.096) |
| BMI (1 increase) | 4.47*10^−8^ | 0.959(0.945–0.974) | 1.75*10^−7^ | 0.961(0.947–0.975) |
| Height (1cm increase) | 0.019 | 0.988(0.979–0.998) | 0.021 | 0.988(0.979–0.998) |
| Overall health (1 level increase) | 3.57*10^−15^ | 0.684(0.622–0.752) | 8.44*10^−15^ | 0.687(0.625–0.755) |
| Systolic BP (1 increase) | 0.414 | 1.001(0.998–1.005) | 0.489 | 1.001(0.998–1.005) |
| Current smoking level (1 increase) | 0.099 | 1.094(0.983–1.218) | 0.079 | 1.1(0.989–1.224) |
| Visuospatial performance (1 error increase) | 9.09*10^−5^ | 1.048(1.024–1.074) | 1.14*10^−4^ | 1.048(1.023–1.073) |
| Male | 5.54*10^−9^ | 1.764(1.458–2.135) | 1.56*10^−8^ | 1.726(1.429–2.086) |
| Currently employed | 0.042 | 0.831(0.696–0.993) | 0.134 | 0.876(0.737–1.041) |
| Sleep duration | 0.135 (group difference) |  | 0.118 (group difference) |  |
| (a) ≤4 h, | - | ref | - | ref |
| (b) 5 h or 6 h, | 0.096 | 0.676(0.427–1.072) | 0.073 | 0.663(0.422–1.04) |
| (c) 7 h or 8 h, | 0.107 | 0.69(0.439–1.084) | 0.062 | 0.657(0.423–1.022) |
| (d) ≤9 h | 0.387 | 0.811(0.506–1.303) | 0.273 | 0.772(0.485–1.227) |
| Alcohol consumption | 0.024 (group difference) |  | 0.028 (group difference) |  |
| (a) 0, | - | Ref | - | Ref |
| (b) 0 < x ≦ 14, | 0.003 | 0.74(0.606–0.905) | 0.004 | 0.745(0.609–0.911) |
| (c) 14 < x ≦ 28 | 0.008 | 0.733(0.584–0.921) | 0.008 | 0.734(0.584–0.922) |
| (d) 28 < x | 0.013 | 0.746(0.591–0.941) | 0.016 | 0.751(0.594–0.948) |
| Non-white (v.s. non-white) | 0.048 | 0.651(0.425–0.996) | 0.087 | 0.694(0.457–1.054) |
| Diabetes | 1.63*10^−6^ | 1.602(1.321–1.942) | 9.87*10^−7^ | 1.618(1.334–1.962) |
| Heart attack | 0.008 | 1.394(1.089–1.786) | 0.004 | 1.43(1.119–1.827) |
| Angina | 0.311 | 1.125(0.896–1.412) | 0.257 | 1.139(0.909–1.428) |
| Stroke | 9.50*10^−6^ | 1.813(1.393–2.359) | 1.26*10^−5^ | 1.805(1.385–2.352) |
| Cancer | 0.183 | 0.87(0.709–1.068) | 0.168 | 0.866(0.705–1.063) |
| Other serious medical conditions | 3.06*10^−5^ | 1.343(1.169–1.543) | 1.20*10^−5^ | 1.363(1.187–1.566) |
| Having driver jobs | 0.845 | 0(0–6.18*10^31^) | 0.849 | 0(0–7.16*10^31^) |

**Supplemental Table 2.** Supplemental analyses’ statistical values of contrasts that are significant in main analyses.

|  | analysis of driving length | | | |  | analysis of nonoccupatonal computer use length | | | |
| --- | --- | --- | --- | --- | --- | --- | --- | --- | --- |
|  | 0h | less than 1h, 1h | 2h,3h | 4h ≤ |  | 0h | less than 1h, 1h | 2h,3h | 4h ≤ |
| main analyses | | | | |  |  |  |  |  |
| P | 3.07*10^−8^ | ref | 2.0*10^−6^ | 0.038 |  | ref | 1.20*10^−9^ | 5.66*10^−4^ | 8.59*10^−3^ |
| HR | 1.544 | ref | 1.574 | 1.525 |  | ref | 0.626 | 0.722 | 0.653 |
| 95%CI | (1.324–1.801) |  | (1.307–1.895) | (1.023–2.274) | |  | (0.538–0.728) | (0.601–0.869) | (0.476–0.897) |
| Supplemental anlalyses based on the age group. | | | | |  |  |  |  |  |
| age ≧60 at baseline | | | | |  |  |  |  |  |
| P | 5.22*10^−7^ | 1.08*10^−7*^ | 4.4*10^−4^ | 0.022 |  | 1.75*10^−7^ | 1.43*10^−8^ | 3.84*10^−4^ | 0.08 |
| HR | 1.539 | ref | 1.544 | 1.698 |  | ref | 0.619 | 0.691 | 0.739 |
| 95%CI | (1.301–1.821) |  | (1.254–1.902) | (1.080–2.671) | |  | (0.525–0.731) | (0.564–0.848) | (0.527–1.037) |
| age <60 at baseline | | | | | |  |  |  |  |
| P | 0.017 | 0.031 | 0.015 | 0.633 |  | 0.047 | 0.048 | 0.71 | 0.026 |
| HR | 1.600 | ref | 1.674 | 1.232 |  | ref | 0.686 | 0.919 | 0.351 |
| 95%CI | (1.107–2.531) |  | (1.107–2.531) | (0.524–2.896) | |  | (0.472–0.997) | (0.588–1.436) | (0.139–0.884) |
| A supplemental analysis including both driving length and nonoccupational computer use length as covariates at once | | | | | | | | | |
| P | 3.35*10^−6^ | 2.41*10^−7*^ | 4.73*10^−6^ | 0.12 |  | 1.36*10^−6^ | 1.19*10^−7^ | 0.003 | 0.014 |
| HR | 1.45 |  | 1.545 | 1.389 |  | ref | 0.66 | 0.753 | 0.672 |
| 95%CI | (1.24–1.696) |  | (1.282–1.861) | (0.918–2.102) | |  | (0.565–0.769) | (0.625–0.907) | (0.489–0.924) |
| A sensitivity analysis excluding subjects with a medical history or cancer, cardiovascular disease, and cognitive/psychiatric illness. | | | | | | | | | |
| P | 0.016 | 0.001* | 0.001 | 0.102 |  | 5.27*10^−4^ | 3.32*10^−4^ | 1.46*10^−3^ | 0.045 |
| HR | 1.331 | Ref | 1.594 | 1.571 |  | ref | 0.677 | 0.623 | 0.578 |
| 95%CI | (1.056–1.678) |  | (1.224–2.076) | (0.915–2.699) | |  | (0.547–0.838) | (0.466–0.834) | (0.339–0.988) |
| Supplemental analyses separating the group of “less than an hour” and 1 h (resulting in 5 categories) | | | | | | | | | |
| Reference is "less than 1h" | | | | |  |  | less than 1h | |  |
| P | 2.98*10^−8^ | 3.65*10^−9*^ | 7.62*10^−7^ | 0.018 |  | 6.06*10^−8^ | 8.10*10^−6^ | 5.65*10^−4^ | 0.009 |
| HR | 1.647 | Ref | 1.686 | 1.641 |  | ref | 0.628 | 0.722 | 0.653 |
| 95%CI | (1.381–1.965) |  | (1.371–2.073) | (1.088–2.476) | |  | (0.512–0.77) | (0.601–0.869) | (0.476–0.897) |
| Reference is 1 h | | | | |  |  | 1h |  |  |
| P | 4.64*10^−5^ | - | 1.62*10^−4^ | 0.08 |  | - | 8.52*10^−8^ | - | - |
| HR | 1.442 | Ref | 1.476 | 1.437 |  | - | 0.625 | - | - |
| 95%CI | (1.209–1.721) |  | (1.206–1.807) | (0.957–2.158) | |  | (0.526–0.742) |  |  |

*P values of existence of any group differences

**References**

Cullen, B., Newby, D., Lee, D., Lyall, D.M., Nevado-Holgado, A.J., Evans, J.J., Pell, J.P., Lovestone, S., and Cavanagh, J. (2018). Cross-sectional and longitudinal analyses of outdoor air pollution exposure and cognitive function in UK Biobank. *Scientific reports* 8**,** 1-14.

Howe, L.J., Lawson, D.J., Davies, N.M., Pourcain, B.S., Lewis, S.J., Smith, G.D., and Hemani, G. (2019). Genetic evidence for assortative mating on alcohol consumption in the UK Biobank. *Nature communications* 10**,** 1-10.

Lourida, I., Hannon, E., Littlejohns, T.J., Langa, K.M., Hyppönen, E., Kuźma, E., and Llewellyn, D.J. (2019). Association of lifestyle and genetic risk with incidence of dementia. *Jama* 322**,** 430-437.

Okbay, A., Beauchamp, J.P., Fontana, M.A., Lee, J.J., Pers, T.H., Rietveld, C.A., Turley, P., Chen, G.B., Emilsson, V., Meddens, S.F., Oskarsson, S., Pickrell, J.K., Thom, K., Timshel, P., De Vlaming, R., Abdellaoui, A., Ahluwalia, T.S., Bacelis, J., Baumbach, C., Bjornsdottir, G., Brandsma, J.H., Pina Concas, M., Derringer, J., Furlotte, N.A., Galesloot, T.E., Girotto, G., Gupta, R., Hall, L.M., Harris, S.E., Hofer, E., Horikoshi, M., Huffman, J.E., Kaasik, K., Kalafati, I.P., Karlsson, R., Kong, A., Lahti, J., Van Der Lee, S.J., Deleeuw, C., Lind, P.A., Lindgren, K.O., Liu, T., Mangino, M., Marten, J., Mihailov, E., Miller, M.B., Van Der Most, P.J., Oldmeadow, C., Payton, A., Pervjakova, N., Peyrot, W.J., Qian, Y., Raitakari, O., Rueedi, R., Salvi, E., Schmidt, B., Schraut, K.E., Shi, J., Smith, A.V., Poot, R.A., St Pourcain, B., Teumer, A., Thorleifsson, G., Verweij, N., Vuckovic, D., Wellmann, J., Westra, H.J., Yang, J., Zhao, W., Zhu, Z., Alizadeh, B.Z., Amin, N., Bakshi, A., Baumeister, S.E., Biino, G., Bonnelykke, K., Boyle, P.A., Campbell, H., Cappuccio, F.P., Davies, G., De Neve, J.E., Deloukas, P., Demuth, I., Ding, J., Eibich, P., Eisele, L., Eklund, N., Evans, D.M., Faul, J.D., Feitosa, M.F., Forstner, A.J., Gandin, I., Gunnarsson, B., Halldorsson, B.V., Harris, T.B., Heath, A.C., Hocking, L.J., Holliday, E.G., Homuth, G., Horan, M.A., et al. (2016). Genome-wide association study identifies 74 loci associated with educational attainment. *Nature* 533**,** 539-542.

Sarkar, S.N., Huang, R.-Q., Logan, S.M., Yi, K.D., Dillon, G.H., and Simpkins, J.W. (2008). Estrogens directly potentiate neuronal L-type Ca2+ channels. *Proceedings of the National Academy of Sciences* 105**,** 15148-15153.

Shen, X., Cox, S.R., Adams, M.J., Howard, D.M., Lawrie, S.M., Ritchie, S.J., Bastin, M.E., Deary, I.J., Mcintosh, A.M., and Whalley, H.C. (2018). Resting-state connectivity and its association with cognitive performance, educational attainment, and household income in the UK Biobank. *Biological Psychiatry: Cognitive Neuroscience and Neuroimaging* 3**,** 878-886.

Takeuchi, H., and Kawashima, R. (2021). Diet and Dementia: A Prospective Study. *Nutrients* 13**,** Article 4500.

Townsend, P. (1987). Deprivation. *Journal of social policy* 16**,** 125-146.

Veldsman, M., Kindalova, P., Husain, M., Kosmidis, I., and Nichols, T.E. (2020). Spatial distribution and cognitive impact of cerebrovascular risk-related white matter hyperintensities. *NeuroImage: Clinical* 28**,** 102405.
